# Identification of biomarkers in diabetic neuropathy: a Mendelian randomization and bioinformatics analysis

**DOI:** 10.3389/fimmu.2026.1793571

**Published:** 2026-04-27

**Authors:** Jiannan Li, Yue Yu, Hongming Zhu, Sibing Chen, Yanxi Liu, Te Zhang

**Affiliations:** 1 Department of Wound Repair, Plastic and Reconstructive Surgery, China-Japan Union Hospital of Jilin University, Changchun, Jilin, China; 2 Department of Endocrinology and Metabolism, China-Japan Union Hospital of Jilin University, Changchun, Jilin, China; 3 Department of Breast Surgery, Second Hospital of Jilin University, Changchun, Jilin, China; 4 Anesthesiology Department, China-Japan Union Hospital of Jilin University, Changchun, Jilin, China

**Keywords:** bioinformatics, biomarkers, diabetic neuropathy, machine learning, Mendelian randomization

## Abstract

**Background:**

This study elucidated several plasma proteins that are causally linked to the risk of diabetic neuropathy (DN), offering novel insights into the protein-mediated DN pathogenesis and potential targets for therapeutic intervention.

**Methods:**

We employed Mendelian randomization (MR) utilizing genome-wide association study (GWAS) data to evaluate the causal effects of 4,907 proteins on DN. We retrieved a high-throughput sequencing dataset (GSE148061, containing 53 DN patients and 53 healthy donors) from the Gene Expression Omnibus (GEO) database to perform differential gene analysis and functional enrichment analysis, aiming to clarify disease pathogenesis. The MR findings were subsequently validated through Bayesian colocalization analysis and cluster identification. We utilized two machine learning algorithms: Least Absolute Shrinkage and Selection Operator (LASSO) and Random Forest (RF). Further, Gene Set Enrichment Analysis (GSEA) and Gene Set Variation Analysis (GSVA) were conducted on these key genes to uncover the underlying molecular mechanisms influencing DN. Finally, we constructed a DN mouse model to validate the diagnostic potential of these identified genes.

**Results:**

We identified seven plasma proteins significantly associated with DN. By integrating Bayesian colocalization, various datasets, and two machine learning methods, we determined that the HSPB1, KRT14, and SFN genes are the most robust diagnostic biomarkers and key mediators. These genes were primarily enriched in the Wnt Signaling Pathway, Regulation of ERK1 and Cascade, Negative Regulation of MAPK Cascade, and Alditol Phosphate Metabolic Process. The expression changes of HSPB1, KRT14, and SFN were further validated using animal models.

**Conclusion:**

This study systematically identified HSPB1, KRT14, and SFN as potential biomarkers for patients with DN.

## Introduction

Diabetic neuropathy (DN) is the most common chronic complication in both type 1 and type 2 diabetes (1). By 2045, 783 million adults worldwide will have diabetes, with up to 350 million developing DN and its comorbidities (2). The treatment of DN primarily focuses on controlling blood glucose, repairing nerves, alleviating oxidative stress, improving microcirculation, and providing symptomatic pain relief (3). However, these treatments cannot completely cure DN and only slow its progression to some extent. Given the high prevalence and associated risk of disability linked to DN, it is imperative to deepen our understanding of its pathogenesis and to explore new therapeutic strategies and novel drug treatments.

Proteins are fundamental components of all cells and tissues in the human body, participating in various biological processes. Plasma proteins play critical roles in numerous biological functions, including signal transduction, transport, growth, repair, and defense against infections. However, these proteins are frequently dysregulated in diseases and serve as significant drug targets (4). Compared to other tissues, blood possesses a complex proteome that mirrors the protein status in tissues. The dynamic nature of blood allows for the monitoring of disease progression both spatially and temporally. Furthermore, the minimally invasive nature of blood sampling enhances its acceptability, which is crucial for its widespread application in clinical assessments (5, 6). Utilizing the largest genome-wide association study (GWAS) summary data to date, Song et al. identified causal relationships between eight plasma proteins and peripheral neuropathy through two-sample Mendelian randomization (MR) and colocalization analysis (7). In a large prospective study, plasma albumin levels were negatively correlated with the incidence of DN, indicating that for every 10 g/L increase in plasma albumin, the hazard ratio (HR) for DN is 0.67 [0.51; 0.88] (8). Complement component C3 is the most abundant protein in serum. A retrospective analysis has confirmed that elevated baseline levels of complement C3 are associated with an increased risk of developing DN in the general population, with a causal relationship established through MR methods (9). The pathogenesis of DN is complex, and the causal relationship between plasma proteins and DN and its pathogenesis have still been studied less. Establishing causal relationships can deepen the understanding of DN mechanisms and guide clinical interventions based on plasma protein profiles for DN. Therefore, identifying key genes involved in the onset of DN through MR analysis is imperative, as well as exploring the molecular changes and functions of these key genes in DN. This understanding is crucial for elucidating the pathogenesis of DN and for informing clinical interventions.

With advancements in interdisciplinary integration and sequencing technology, researchers have achieved a deeper understanding of the pathophysiological mechanisms underlying diseases by leveraging computer science and sequencing data. MR is a causal inference method that evaluates the causal effects of various exposure factors (such as diseases, gene/protein expression, metabolites, and gut microbiota) on clinical outcomes through genetic variations (10). This method significantly mitigates the influence of most confounding factors on outcomes, making it widely applicable (11).

In this study, we systematically screened key proteins significantly associated with DN by integrating various methods, including Mendelian randomization, Bayesian colocalization analysis, RNA-seq differential analysis, machine learning, Gene Set Enrichment Analysis (GSEA), Gene Set Variation Analysis (GSVA), and *in vivo* animal models. This multifaceted approach provides new insights into the molecular mechanisms underlying DN and establishes a foundation for future clinical applications.

## Methods

### Study design

**Figure 1:**
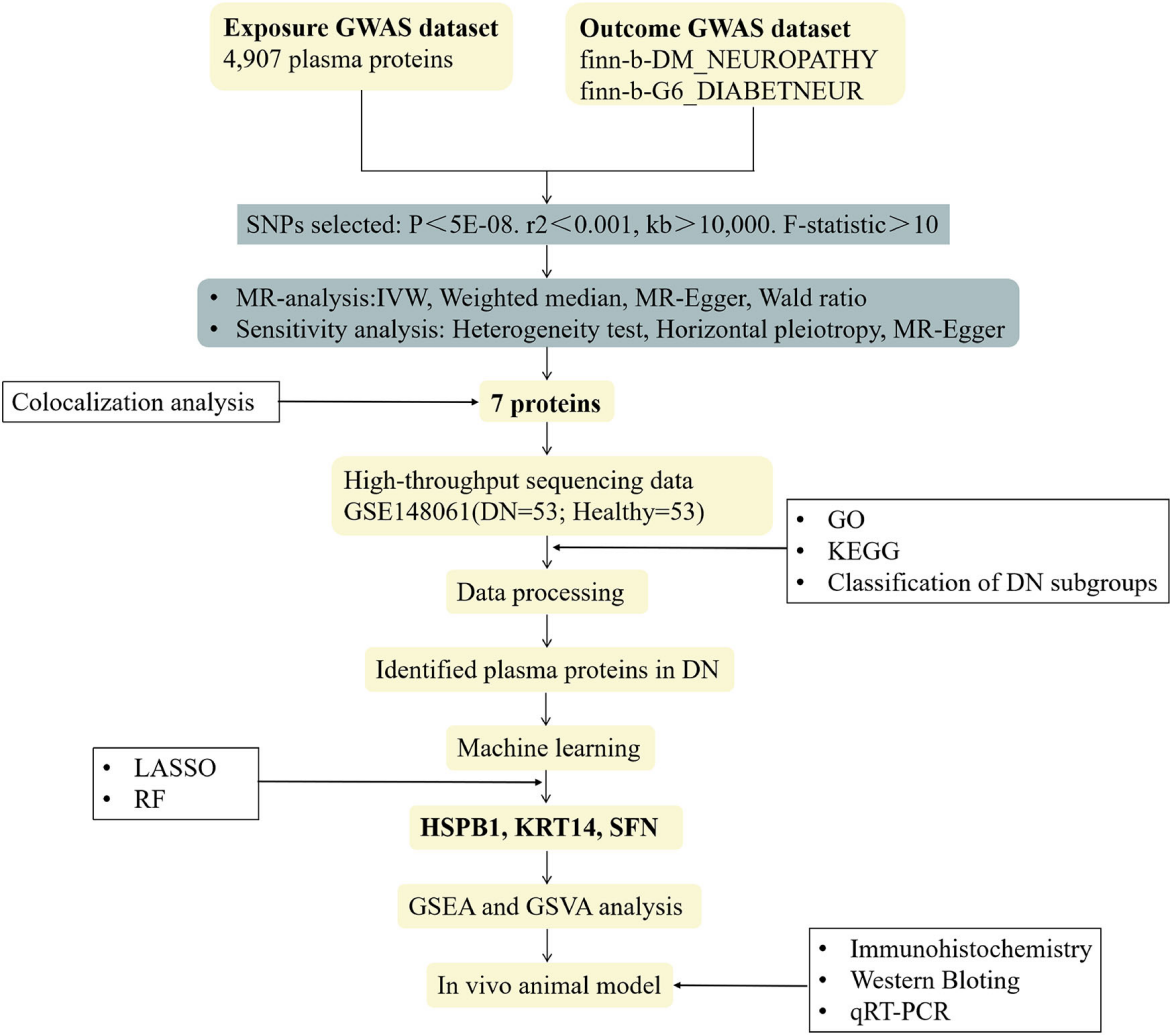
The flow chart. Using 4,907 plasma proteins as the exposure GWAS data and finn-b-DM_NEUROPATHY and finn-b-G6_DIABETNEUR as the outcome GWAS data, SNPs are filtered according to predefined criteria, followed by MR analysis and sensitivity tests. Combined with Bayesian colocalization analysis, seven plasma proteins significantly associated with DN are identified. Subsequently, the GSE148061 dataset (53 DN and 53 healthy) is utilized to perform differential gene screening, functional enrichment analysis and DN subgroup classification. By taking the intersection of results from the LASSO and RF machine learning algorithms, HSPB1, KRT14 and SFN are defined as the core key genes. GSEA and GSVA are then conducted to elucidate the molecular pathways through which these genes regulate DN pathogenesis. Finally, a spontaneous diabetic mouse model of DN is established, and immunohistochemistry, Western Blotting and qRT-PCR are employed to verify the expression of these three genes in relevant mouse tissues, thus confirming their potential as DN biomarkers.

**Figure 1** illustrates the comprehensive screening and analysis process employed in this study. Initially, we selected data from a large-scale GWAS focused on the plasma proteome to develop an MR analysis framework linking the proteome to DN, thereby identifying plasma proteins causally associated with DN. In the subsequent step, we further evaluated the initially causal associations using heterogeneity and pleiotropy tests. We then employed Bayesian colocalization methods to determine whether the plasma proteins and DN share causal variants. Following this, we conducted Gene Ontology (GO) and Kyoto Encyclopedia of Genes and Genomes (KEGG) enrichment analyses on differentially expressed proteins using the GSE148061 dataset, which further validated the MR results. In the penultimate step, we integrated two machine learning approaches—Least Absolute Shrinkage and Selection Operator (LASSO) regression and Random Forest (RF)—to identify the final key proteins. The relevant signaling pathways associated with these key proteins affecting DN were analyzed using GSVA and GSEA. Finally, we constructed a spontaneous diabetic mouse model to verify the levels of the identified proteins through molecular biology techniques.

The summary-level data used in this study are publicly available and de-identified. The GWAS included have been approved by their respective institutions.

### Data source

All data analyzed in this study were derived from the summary-level data presented in **Supplementary Table 1**. All participants were of European descent. The plasma proteomics data were sourced from the GWAS conducted by deCODE Health (https://www.decode.com/summarydata/). The deCODE Health study employed an aptamer-based multiplex approach, specifically the SOMAscan V4 assay, to assess 4,907 plasma proteins in a cohort of 35,559 Icelanders (12). The high-throughput sequencing dataset for DN was obtained from the Gene Expression Omnibus (GEO) (https://www.ncbi.nlm.nih.gov/geo/). The search terms were: (Diabetic neuropathy) AND (Homo sapiens). Each GEO dataset was required to fulfill the following criteria: (1) complete clinical protein expression data; (2) inclusion of studies with both DN patients and healthy controls; (3) a sample size greater than three in each group. Ultimately, the GSE148061 dataset was selected for further validation of the MR results. This dataset, submitted by Guo K et al. and publicly available since August 24, 2020 (13), was generated using high-throughput sequencing for genome-wide DNA methylation and gene expression profiling. It comprises 53 cases of DN and 53 healthy subjects.

Additionally, this study utilized the IEU Open GWAS database (https://gwas.mrcieu.ac.uk/), with DN as the outcome (GWAS ID: finn-b-DM_NEUROPATHY; finn-b-G6_DIABETNEUR). The characteristics of data used in this study are shown in **Table 1**.

**Table 1:** Characteristics of data in this study.

Outcomes	GWAS ID	Year	Category	Sub category	Population	Sex	ncase	ncontrol	Number of SNPs	Unit	Author	Consortium	Ontology	Build	Note
Diabetic neuropathy	finn-b-DM_NEUROPATHY	2021	Binary	NA	European	Males and Females	1415	162201	16380195	NA	NA	NA	NA	HG19/GRCh37	DM_NEUROPATHY
Diabetic neuropathy	finn-b-G6_DIABETNEUR	2021	Binary	NA	European	Males and Females	1419	195047	16380416	NA	NA	NA	NA	HG19/GRCh37	G6_DIABETNEUR

### MR analysis

The genetic instruments utilized in MR were selected from protein quantitative trait loci (pQTL), with the platform ID for each protein corresponding to its gene symbol. pQTL refers to the genetic variation sites related to the protein expression level. The criteria for selecting instrumental variables and proteins are as follows: (1) Single nucleotide polymorphisms (SNPs) used as genetic tools for each plasma protein were selected based on the significance level of the whole genome (P<5×10^-8^), P<5×10^–8^ was to control genome-wide false positives and ensure the true association between SNPS and exposure; (2) To ensure that we only select for unique SNPs, we used R^2^<0.001 to eliminate linkage disequilibrium (LD) and ensure the independence of SNPS; (3) To ensure the independence of SNPs, SNPs with LDR^2^>0.001 within the range of 10Mb are excluded; (4) The strength of the genetic instruments was estimated using R^2^ and the F-statistic (R^2^=2×EAF×(1-EAF)×beta^2^, F=R^2^×(N-2)/(1-R^2^)) (14), where R^2^ represents the proportion of variability in protein levels explained by each genetic instrument. For proteins that appeared multiple times in the study, R^2^ and the largest protein were selected.

The MR analysis was conducted using the “TwoSampleMR” package. For plasma proteins with a single instrumental variable (IV), the Wald ratio method was employed to estimate the change in the log probability of developing DN per one standard deviation (SD) increase in plasma protein levels indicated by the IV (15). For proteins with multiple IVs, the inverse-variance weighted (IVW) method was utilized to estimate the MR effect. Heterogeneity testing was performed using Cochran’s Q statistic to assess the heterogeneity of the genetic instruments. Results from MR-Egger were adopted only when the intercept indicated the presence of horizontal pleiotropy.

### Bayesian colocalization analysis

Bayesian colocalization analysis is a statistical method used to determine whether two or more phenotypes are driven by the same causal variation in the same region (16). The purpose of Bayesian colocalization analysis is to enhance the findings of genetic studies by identifying shared genetic variants associated with both exposure and outcome (16). This research method is particularly advantageous for assessing exposures such as protein levels and gene expression, especially when MR analysis focuses on specific gene regions. We utilized the ‘coloc’ package (https://github.com/ZTJLU/diabetic-neuropathy-data.git) for colocalization analysis to identify associations between known proteins and DN. Throughout the analysis process, we adhered to the default parameters of the software package, which include p1 = 1×10^-4^ (the prior probability of SNP association with protein), p2 = 1×10^-4^ (the prior probability of SNP association with DN), and p12 = 1×10^-5^ (the prior probability of SNP association with both protein and DN). We calculated the posterior probability (PP) for each hypothesis, and when the posterior probability of hypothesis 4 (PPH4) is ≥ 0.8, it is classified as High, indicating strong evidence of colocalization between the two signals. When PPH4 is < 0.8, it is considered as Low, indicating that the possibility of colocalization between the two signals is relatively small.

### Differentially expressed gene screening and identification

The DEGs in the GSE148061 dataset were identified using the “Limma” package of R software (17). The screening parameters of DEGs were set as fold change (FC) > 1.5 and false discovery rate (FDR) < 0.05. The volcano plot of DEGs was further generated using the “ggplot2” package of R software.

To determine the main biological processes and functions enriched by DEGs, we performed GO and KEGG analyses on DEGs using the “clusterprofiler” package (18) of R software. p<0.05 indicates a statistically significant difference.

To determine the expression of circulating proteins identified by MR in the GSE148061 dataset, boxplots of the circulating proteins were plotted using the “ggpubr” R package.

### Consensus clustering analysis

The R package ConsensusClusterPlus (19) was used for cluster classification, DN patients were stratified into different subgroups, and the optimal clustering is evaluated through cumulative distribution function (CDF) and consensus matrix. The R package “limma” was used again to analyze the differential genes of DN subtypes in the data set, with |log_2_FC|>1 and P < 0.05 as the differential gene-screening criteria. DEGs with |log_2_FC|>4 and P<0.05 were considered upregulated, whereas those with |log_2_FC|<-3 and P<0.05 were considered downregulated. The results are displayed using a volcano map. Volcano plots and box plots were drawn through the “ggplot2” and “ggpubr” packages of the R software.

### Machine learning for key gene screening

We applied two commonly used machine learning methods, LASSO and RF, to screen for relatively more important feature genes. LASSO belongs to the family of linear regression models and demonstrates superiority over regression analysis in evaluating high-dimensional data, enabling data dimensionality reduction. Using the glmnet package (20), 10-fold cross-validation was selected, and the penalty parameter was utilized to achieve data dimensionality reduction in LASSO analysis. In recent years, LASSO regression analysis has been widely used in studies screening for disease-related diagnostic or prognostic factors (21). The RF algorithm is also a supervised machine learning method (22) and was employed in this study to rank the importance of DN-related genes, with predictive performance assessed through 10-fold cross-validation. For the gene sets obtained from these two machine learning methods, the intersection was taken to ultimately identify our key genes. The screened key genes were validated using datasets from cluster 1, cluster 2, normal, and DN, and box plots were generated using the “ggpubr” package.

### GSVA analysis

GSVA changes the analysis object from genes to gene sets and conducts difference analysis at the gene set (pathway) level (23). Utilizing the GSVA package (23) in R software, the enrichment score for each sample within the gene set was calculated. The rank of the gene row was predefined, and the gene expression profile was employed. The subset c2.cp.kegg.v7.4.symbols.gmt was downloaded from the Molecular Signatures Database. To evaluate the relevant pathways and molecular mechanisms, the minimum gene set size was established at 5, while the maximum gene set size was set to 5000. The enrichment score for each sample across each gene set was computed, resulting in the generation of an enrichment score matrix. The differences in GSVA scores between samples in the high-expression and low-expression groups were analyzed using the limma software package. |t|>2, P<0.05 were used as screening conditions. If t>0, the pathway was considered to be activated in the high expression group, and t<0, the pathway was considered to be activated in the low expression group. This analysis was completed using SangerBox online software (http://sangerbox.com), and bidirectional bar charts were generated on the Weshengxin online plotting platform.

### GSEA analysis

GSEA involves comparing gene expression data with a pre-defined gene set to capture the collective modulation patterns of the gene set, thereby providing a more comprehensive biological interpretation (24). This study utilized the Molecular Signatures Database (http://www.gsea-msigdb.org/gsea/downloads.jsp) to download the C2.Cp.Kegg.V7.4 symbols and the GMT collections for evaluating pathways and molecular mechanisms. The GSEA analysis of the HSPB1, KRT14, SFN genes were conducted using SangerBox online software (http://sangerbox.com) to determine the differential biological signaling pathways associated with the expression level of the HSPB1, KRT14, SFN genes.

### Animal feeding and sourcing

A total of six SPF-grade male C57BL/KsJ-db/db (db) mice aged 12 weeks, along with 6 wild-type mice of the same sex, age, and genetic background, were purchased from Cyagen (Suzhou) Biotechnology Co., Ltd. The mice were housed in the SPF-level animal facility of Jilin University, where the room was maintained at a temperature of (24 ± 2)°C and a humidity of (50 ± 10)%, with a 12 h/12 h artificial light/dark cycle, and provided with free access to food and water. All animal experiments in this study were approved by the Animal Ethics and Welfare Committee of Wuchuang Biotechnology (Shanghai, China) Co., LTD (Approval No.: WTPZ20241122004). Blood was collected from the tail vein of the mice, and fasting blood glucose and body weight were measured using a portable glucose meter. After determining the mechanical pain threshold and thermal pain threshold, the mice were euthanized, and the dorsal root ganglia and spinal cord tissues were harvested.

### Mechanical pain threshold testing

Using von Frey filaments, the mechanical allodynia of the hind paw plantar surface in mice was assessed according to the Up-down method. Mice were placed in transparent single cages on the testing rack in advance to acclimate to the testing environment. Then, the stimulation pen was held to apply a consistent force perpendicularly upward to the hind paw plantar surface of the mice until the filament bent into an “S” shape. The test started with a stimulation intensity of 0.4 g, each lasting 6–8 seconds, with a 1-minute interval between tests. If a paw withdrawal reflex was observed upon stimulation of the hind paw, it was recorded as “×”, and a smaller-sized stimulation pen was used for the next test. If no response was observed, it was recorded as “○”, and a larger-sized stimulation pen was used for the next test, and so on. The test was terminated after four additional tests following the first occurrence of the paw withdrawal reflex. Identify the corresponding coefficients from the recorded spectra and the coefficient corresponding to the last test, and substitute them into the logarithmic formula for stimulus force to calculate the 50% withdrawal threshold (25).

### Thermal pain threshold testing

Measure the paw withdrawal latency (PWL) in mice using a hot plate analgesia meter. Set the hot plate analgesia meter to 55 °C, place a transparent glass around the hot plate, and position the mouse with its forepaws on the ground and hind paws suspended over the hot plate. Release the mouse and start timing. A positive response is indicated by the mouse licking its hind paws, jumping in place, or showing obvious leg retraction. Stop timing and record the time as the recorded response latency (25).

### Immunohistochemical staining

Paraffin sections of dorsal root ganglion tissue were dewaxed in xylene and gradient ethanol, followed by antigen retrieval using citrate sodium antigen retrieval buffer. The sections were then incubated in 3% hydrogen peroxide to block endogenous peroxidase activity, and blocked with bovine serum albumin (BSA). Primary antibodies were applied and incubated overnight at 4°C. On the second day, the sections were washed with phosphate-buffered saline (PBS), followed by incubation with secondary antibodies at room temperature for 50 minutes. After PBS washing, the sections were developed with diaminobenzidine (DAB) chromogen solution, and the reaction was stopped. Hematoxylin was used to counterstain the nuclei for immunohistochemistry. Images were captured under a microscope and analyzed using ImageJ software. The distribution density of positive cells was calculated using the formula “mean gray value = integrated gray value/total area of the field of view”.

### Western blotting

The spinal cord tissue samples were lysed using radio immunoprecipitation assay (RIPA) lysis buffer, and the protein concentration was determined using the bicinchoninic acid (BCA) kit. The denatured proteins were loaded for sodium dodecyl sulfate polyacrylamide gel electrophoresis (SDS-PAGE) gel electrophoresis and transferred to a membrane. After transfer, the membrane was blocked with blocking buffer, incubated with primary antibodies, and developed using enhanced chemiluminescence (ECL). Image J software was used for quantitative analysis of the bands, and the relative expression levels of the target proteins were normalized to β-actin.

### qRT-PCR

Total RNA was extracted using TRIzol reagent (Invitrogen), and cDNA was synthesized using the PrimeScript™ RT Reagent Kit with gDNA Eraser (TaKaRa, Japan). The RNA levels were detected by quantitative real-time PCR (qRT-PCR) using the SYBR Green PCR Kit (TaKaRa, Japan). β-actin was used as the internal reference, and the relative gene expression was calculated using the 2^-ΔΔCt^ method. Each group was maintained in triplicate. The primer sequences are shown in **Table 2**.

**Table 2:** Forward and reverse sequences of primers.

Primer	Sequence (5′ to 3′)
HSPB1-F	TGGACCCCACCCAAGTTTC
HSPB1-R	CGGCAGTCTCATCGGATTTT
KRT14-F	CAGTCCCAGCTCAGCATGAA
KRT14-R	TGGGAAGATGAAAGGTGGGC
SFN-F	TCCACTACGAGATCGCCAACAG
SFN-R	GTGTCAGGTTGTCTCGCAGCA
β-actin-F	GGCTGTATTCCCCTCCATCG
β-actin-R	TGTACCGTAACAATGGTTGACC

### Statistical analysis

The R software (version 4.1.1) was utilized for data analysis and generation of figures and plots. Data are expressed as mean ± SD. Statistical analysis was carried out with GraphPad Prism. Boxplots were created using the ggplot2 package in R. Differences between two groups were compared using the Student’s t-test, with P<0.05 considered statistically significant, * indicates P<0.05; ** indicates P<0.01; *** indicates P<0.001.

## Results

### Proteome-wide MR and colocalization analysis

**Figure 2:**
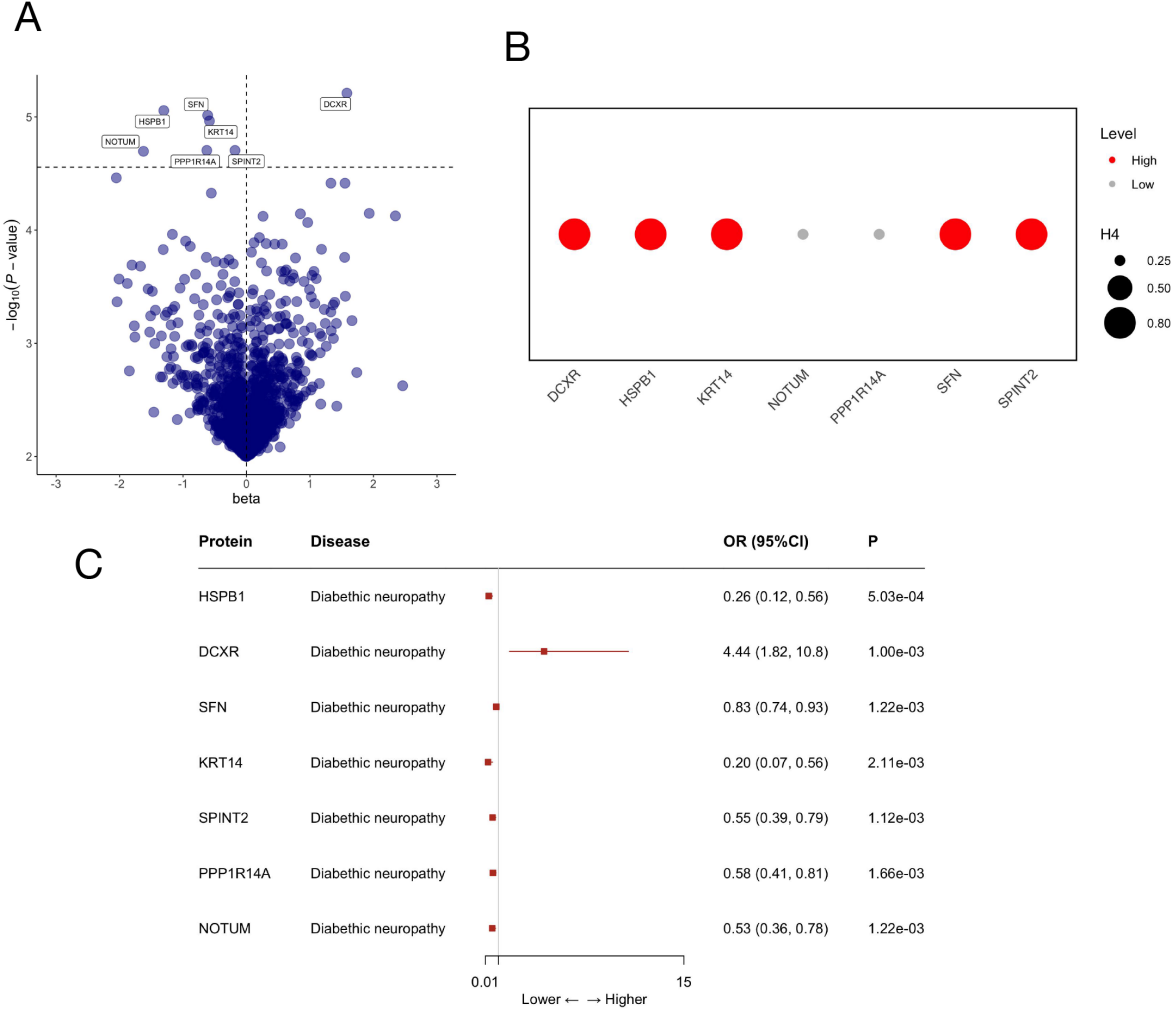
Clarifies the directional association of seven candidate genes with DN risk. **(A)** Manhattan plot showing the causal effect estimates (beta) and statistical significance (−log_10_(P)) of 4,907 plasma proteins for DN; the horizontal dashed line indicates the genome-wide significance threshold (P<5×10^-8^), with the seven candidate proteins labeled. **(B)** Colocalization analysis results for the seven proteins, where circle size represents the posterior probability for shared causal variants (PPH4), and color intensity denotes the level of colocalization evidence (High/Low). **(C)** Forest plot displaying the OR and 95% CI for the associations of the seven proteins with DN, with red squares indicating effect sizes and horizontal lines representing 95% CI; statistical significance is indicated by P values.

Using GWAS ID: finn-b-DM_NEUROPATHY as the outcome data and plasma proteomics data as the exposure data, MR analysis identified seven plasma proteins associated with DN (**Figure 2A**, **Supplementary Table 1**, **S3**). These proteins include: Heat Shock Protein Family B (Small) Member 1 (HSPB1), Dicarbonyl And L-Xylulose Reductase (DCXR), Stratifin (SFN), Keratin 14 (KRT14), Serine Peptidase Inhibitor, Kunitz Type 2 (SPINT2), Protein Phosphatase 1 Regulatory Inhibitor Subunit 14A (PPP1R14A), Notum, Palmitoleoyl-Protein Carboxylesterase (NOTUM). Specifically, HSPB1 (OR = 0.26; 95% CI = 0.12-0.56; P = 5.03×10^-4^), SFN (OR = 0.83; 95% CI = 0.74-0.93; P = 1.22×10^-3^), KRT14 (OR = 0.20; 95% CI = 0.07-0.56; P = 2.11×10^-3^), SPINT2 (OR = 0.55; 95% CI = 0.39-0.79; P = 1.12×10^-3^), PPP1R14A (OR = 0.58; 95% CI = 0.41-0.81; P = 1.66×10^-3^), and NOTUM (OR = 0.53; 95% CI = 0.36-0.78; P = 1.22×10^-3^) were associated with a reduced risk of DN, while DCXR (OR = 4.44; 95% CI = 1.82-10.8; P = 1.00×10^-3^) was associated with an increased risk of DN. Since all the seven circulating proteins identified by MR had only 1–2 instrumental SNPs, and the number of SNPs required for heterogeneity and pleiotropy analysis, sensitivity analysis confirmed that the robustness of MR analysis was limited in this part (**Supplementary Table 4**, **Supplementary Table 5**). To improve the reliability of MR causal inference, we based our analysis on the original exposure data and replaced the outcome data with an independent GWAS dataset (finn-b-G6_DIABETNEUR) to perform comprehensive external validation of the seven candidate genes initially screened by MR. The results confirmed that four of these genes (SPINT2, NOTUM, PPP1R14A, DCXR) have stable causal associations with DN, while the remaining three genes did not show significant associations in this independent dataset (**Supplementary Table 2**). Next, we conducted Bayesian colocalization analysis on seven circulating proteins in relation to DN (**Figure 2**). A high degree of support for co-localization evidence was observed among HSPB1, SFN, KRT14, SPINT2, DCXR and DN. The possibility of colocalization among PPP1R14A, NOTUM and DN is relatively low. Therefore, we included these 5 genes in subsequent studies to further explore their roles as pathological mediators and potential therapeutic targets in DN.

### Validate MR results with GSE148061 dataset

**Figure 3:**
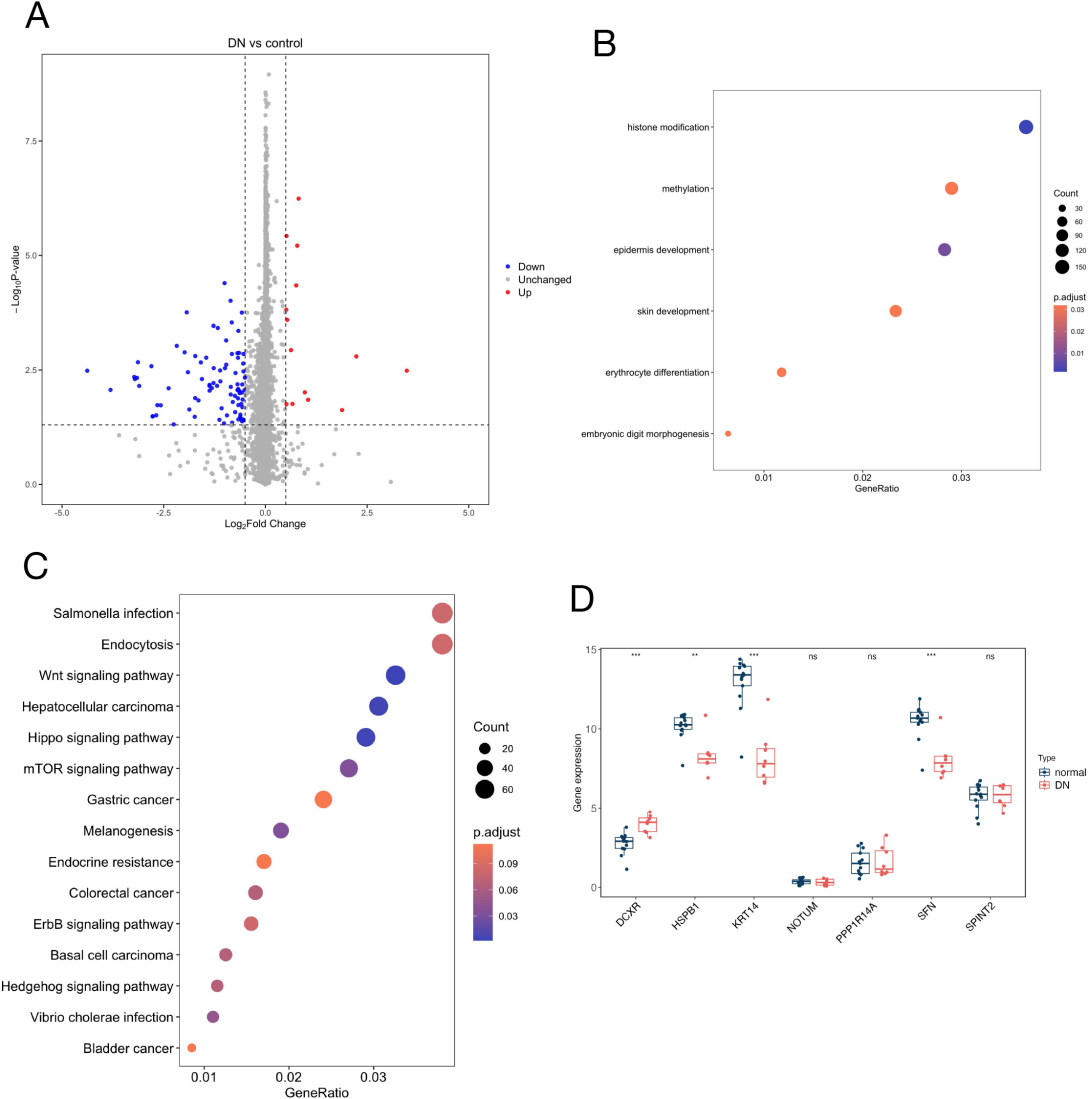
Validation of causal plasma proteins for diabetic neuropathy via the GSE148061 dataset. **(A)** Volcano plot showing DEGs between DN and normal control samples in the GSE148061 cohort, with red indicating upregulated genes, blue indicating downregulated genes, and gray indicating non-significant genes; **(B)** Bubble plot displaying the top enriched GO biological processes for DEGs, with the x-axis representing gene ratio and the color gradient representing adjusted P-values; **(C)** Bubble plot illustrating the top enriched KEGG pathways for DEGs, with the x-axis representing gene ratio and the color gradient representing adjusted P-values; **(D)** Box plots comparing the expression levels of the seven key proteins identified by MR between DN and normal control groups in the GSE148061 dataset. **P<0.01, ***P<0.001 indicating significant differences.

Since sensitivity analysis was limited by 1–2 instrumentalb SNPs in the protein identified by MR, we further utilized the dataset to validate the MR results. Through the application of the ‘Limma’ package, a differential analysis of the GSE148061 dataset identified a total of 1264 DEGs, comprising 1057 upregulated and 207 downregulated genes (**Figure 3**, **Supplementary Table 6**). Functional enrichment analysis of these DEGs was conducted using GO and KEGG methodologies, revealing that the DEGs were predominantly enriched in processes related to histone modification, methylation, epidermis development, and skin development (**Figure 3**). The KEGG pathway analysis indicated that the DEGs were primarily associated with the Wnt signaling pathway, hepatocellular carcinoma, Hippo signaling pathway, mTOR signaling pathway, Salmonella infection, and endocytosis (**Figure 3**). These findings suggest that DEGs are closely linked to epigenetics and DN-related signaling pathways. The box plot analysis of the circulating proteins identified by the MR method (**Figure 3**) demonstrated that, in comparison to the normal group samples, the high-risk gene DCXR was significantly overexpressed in the DN group, while the low-risk genes HSPB1, KRT14, and SFN were significantly underexpressed in the GSE148061 dataset.

### DN subgroup classification and DEG expression in GSE148061

**Figure 4:**
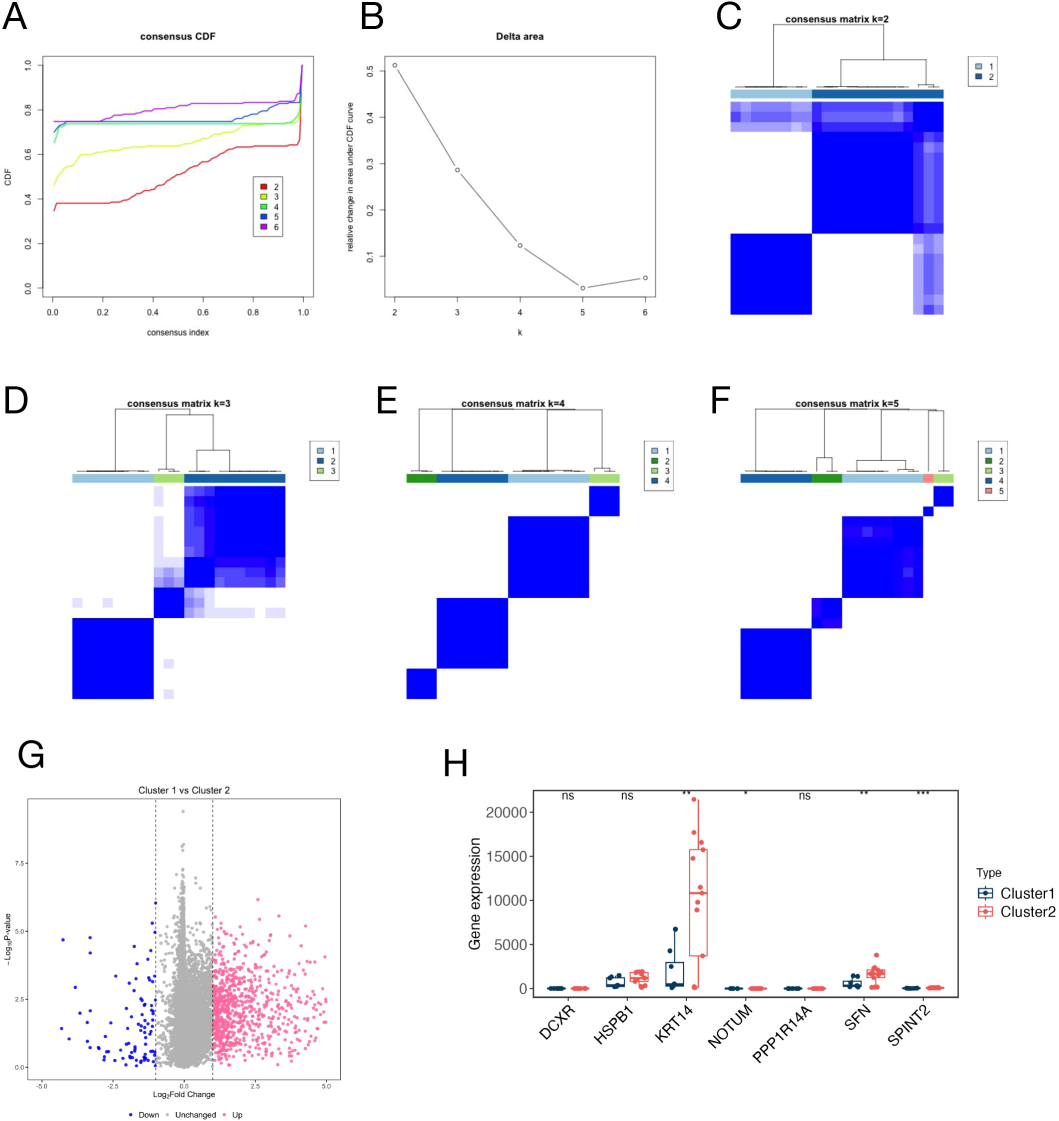
Identification of robust molecular subtypes of diabetic neuropathy via consensus clustering in the GSE148061 dataset. **(A)** Consensus CDF curves for clustering solutions with k values ranging from 2 to 6, illustrating the stability of cluster assignments; **(B)** Delta area plot showing the relative change in the area under the CDF curve, used to determine the optimal number of clusters (k); **(C–F)** Consensus clustering matrices for k=2, 3, 4, and 5, respectively, with color intensity representing the consensus score (darker blue indicates higher co-clustering probability); **(G)** Volcano plot displaying DEGs between Cluster 1 and Cluster 2, with the x-axis representing log₂(fold change) and the y-axis representing -log₁₀(P-value); red dots indicate upregulated DEGs, blue dots indicate downregulated DEGs, and gray dots indicate non-significant DEGs; **(H)** Box plots comparing the expression levels of seven key genes (DCXR, HSPB1, KRT14, NOTUM, PPP1R14A, SFN, SPINT2) between Cluster 1 and Cluster 2. *P<0.05, **P<0.01, ***P<0.001 indicating significant differences.

Furthermore, we employed a co-clustering identification method to explore different subtypes of DN by evaluating the expression differences within the GSE148061 dataset. Further analysis was conducted on the expression levels of circulating proteins identified by MR in the new subtypes. **Figure 4** illustrates the CDF at different K values, where a smaller decline slope in the CDF indicates a more reliable choice for the K value. Additionally, **Figure 4** reflected the relative changes in the area under the CDF curve for K compared to K-1. When K = 3, the increase in the area under the curve was relatively small, suggesting that K = 2 may be a more appropriate choice to ensure clustering stability. The matrix heatmap further demonstrated that the distinction between the two sample clusters was more pronounced at K = 2 (**Figure 4C**). Furthermore, we plotted a volcano plot to illustrate the distribution of DEGs between the two clusters, cluster 1 versus cluster 2, revealing 1,057 upregulated and 207 downregulated genes (**Figure 4**). In the molecular subtype stratification analysis (**Figure 4**), KRT14, NOTUM, SFN, and SPINT2 were significantly higher in Cluster 2 than in Cluster 1, while DCXR, HSPB1, and PPP1R14A showed no significant expression differences between the two subtypes. These results further reflect the molecular heterogeneity of DN and highlight the need to integrate multidimensional information to screen for core candidate genes. Additionally, the distinct expression patterns across molecular subgroups provide important context for interpreting the potential roles of risk-reducing genes such as HSPB1, KRT14, and SFN, suggesting their involvement in tissue-specific or context-dependent regulatory processes in DN.

### Key gene identification by machine learning

**Figure 5:**
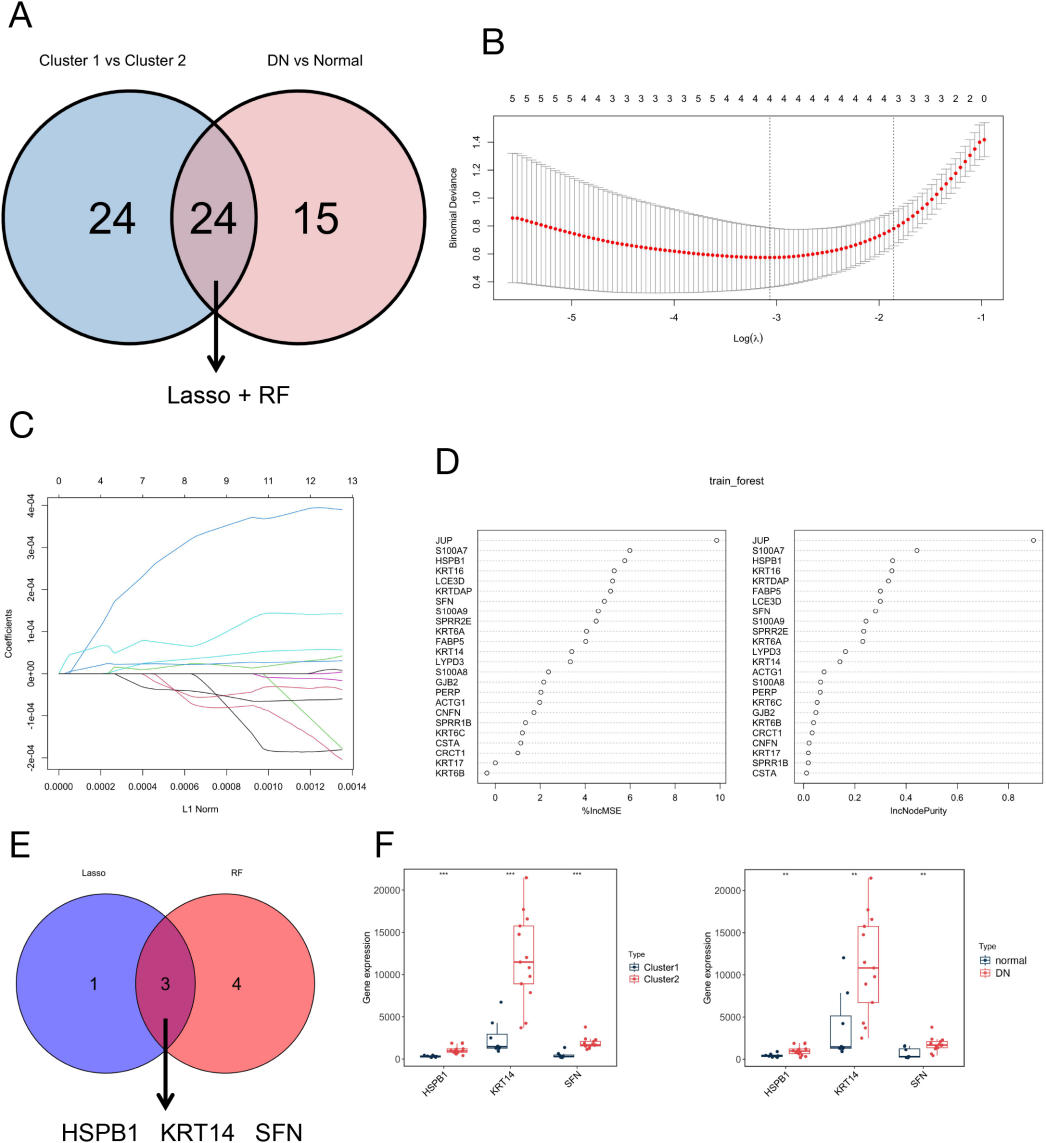
Identification of HSP1, KRT14, and SFN as core biomarkers for DN via integrated machine learning analysis. **(A)** Venn diagram showing the intersection of DEGs between Cluster 1 vs. Cluster 2 and DN vs. normal groups, with candidate genes for subsequent machine learning screening indicated by the arrow; **(B)** LASSO coefficient profiles of the candidate genes, with the x-axis representing the log(λ) value and the y-axis representing the binomial deviance; **(C)** Ten cross‐validations of adjusted parameter selection in the LASSO model; **(D)** RF algorithm-based feature selection and gene importance ranking for identifying key DN-related genes; **(E)** Venn diagram illustrating the overlapping genes identified by LASSO regression and RF algorithms, resulting in three core key genes (HSPB1, KRT14, SFN); **(F)** Box plots showing the expression levels of HSPB1, KRT14, and SFN in Cluster 1 vs. Cluster 2 and DN vs. normal datasets.

We combined cluster 1 versus cluster 2 and normal versus DN, utilizing two common machine learning methods, LASSO and RF, to identify 24 key differential genes (**Figure 5**). The LASSO algorithm identified four key genes (**Figure 5B**), while the RF algorithm ranked the key genes by importance, screening seven key genes (**Figure 5**). By intersecting the results from both machine learning methods, we further refined our selection to three key genes: HSPB1, KRT14, and SFN (**Figure 5**). We then examined the expression levels of these three genes in cluster 1, cluster 2, Normal, and DN samples. Our comparison revealed that HSPB1, KRT14, and SFN were highly expressed in the DN and cluster 2 groups (**Figure 5**). Consequently, these three genes would be the focus of our further research.

### GSEA and GSVA of core genes

**Figure 6:**
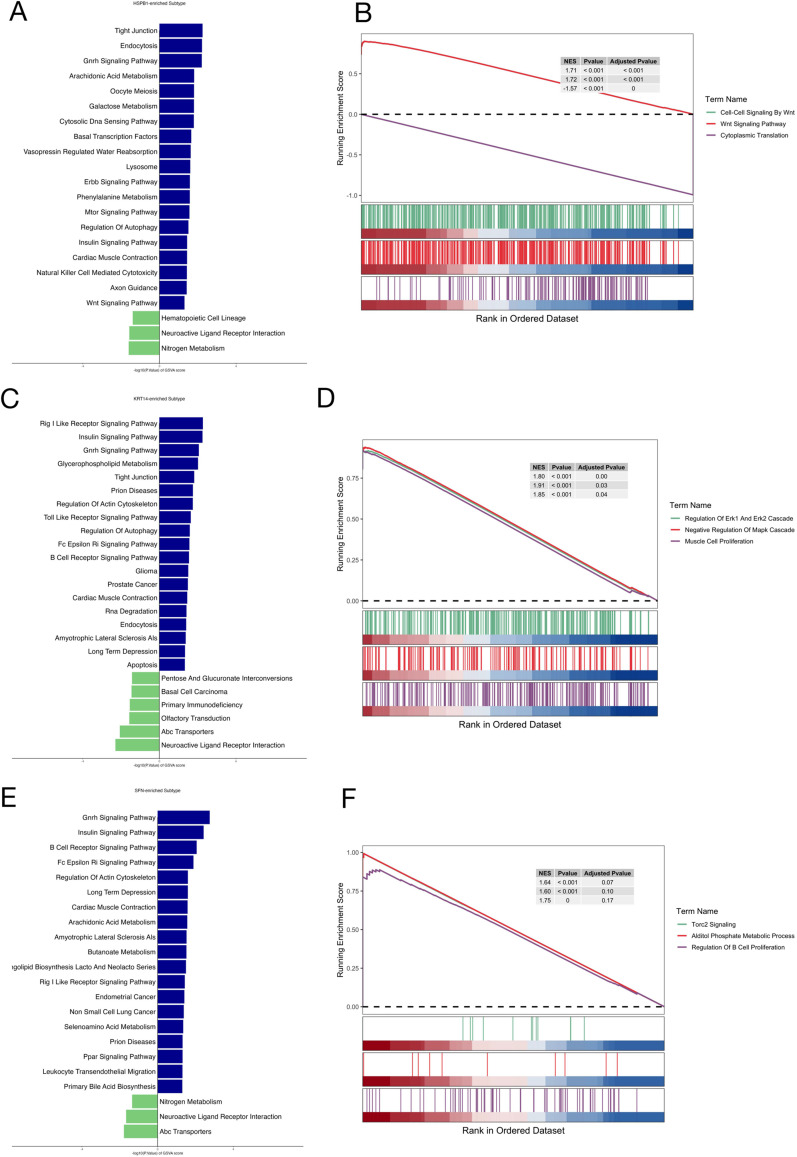
HSPB1, KRT14, and SFN drive distinct pathway enrichments in DN revealed by GSVA and GSEA. **(A, C, E)** GSVA showing the enrichment profiles of HSPB1, KRT14, and SFN across hallmark signaling pathways in DN; **(B, D, F)** GSEA illustrating the enrichment of these core genes with key biological pathways implicated in DN pathogenesis.

Next, we analyzed the signaling pathways of the HSPB1, KRT14, and SFN genes using GSEA and GSVA to investigate their potential molecular mechanisms influencing DN. The GSVA results presented in **Figure 6** indicated that high expression levels of HSPB1 primarily activated processes such as the Wnt Signaling Pathway and galactose metabolism. The GSEA results illustrated in **Figure 6** demonstrate that HSPB1 was mainly enriched in the Wnt Signaling Pathway. These results suggest that HSPB1 may affect the progression of DN through the Wnt Signaling Pathway. Furthermore, the GSVA results shown in **Figure 6** revealed that high expression of KRT14 primarily activated processes associated with apoptosis and the insulin signaling pathway. The GSEA results depicted in **Figure 6** indicate that KRT14 was predominantly enriched in the regulation of ERK1 and cascade, negative regulation of MAPK cascade, and muscle cell proliferation. These results indicate that KRT14 may affect the progression of DN through ERK1 and MAPK cascade. The GSVA results presented in **Figure 6** showed that high expression levels of SFN mainly activated processes related to the insulin signaling pathway and the B cell receptor signaling pathway. Finally, the GSEA results illustrated in **Figure 6** indicated that SFN was primarily enriched in the alditol phosphate metabolic process and the regulation of B cell proliferation. SFN may be associated with DN pathogenesis through the Insulin Signaling Pathway and the B Cell Receptor Signaling Pathway.

### *In vivo* validation in DN mice

**Figure 7:**
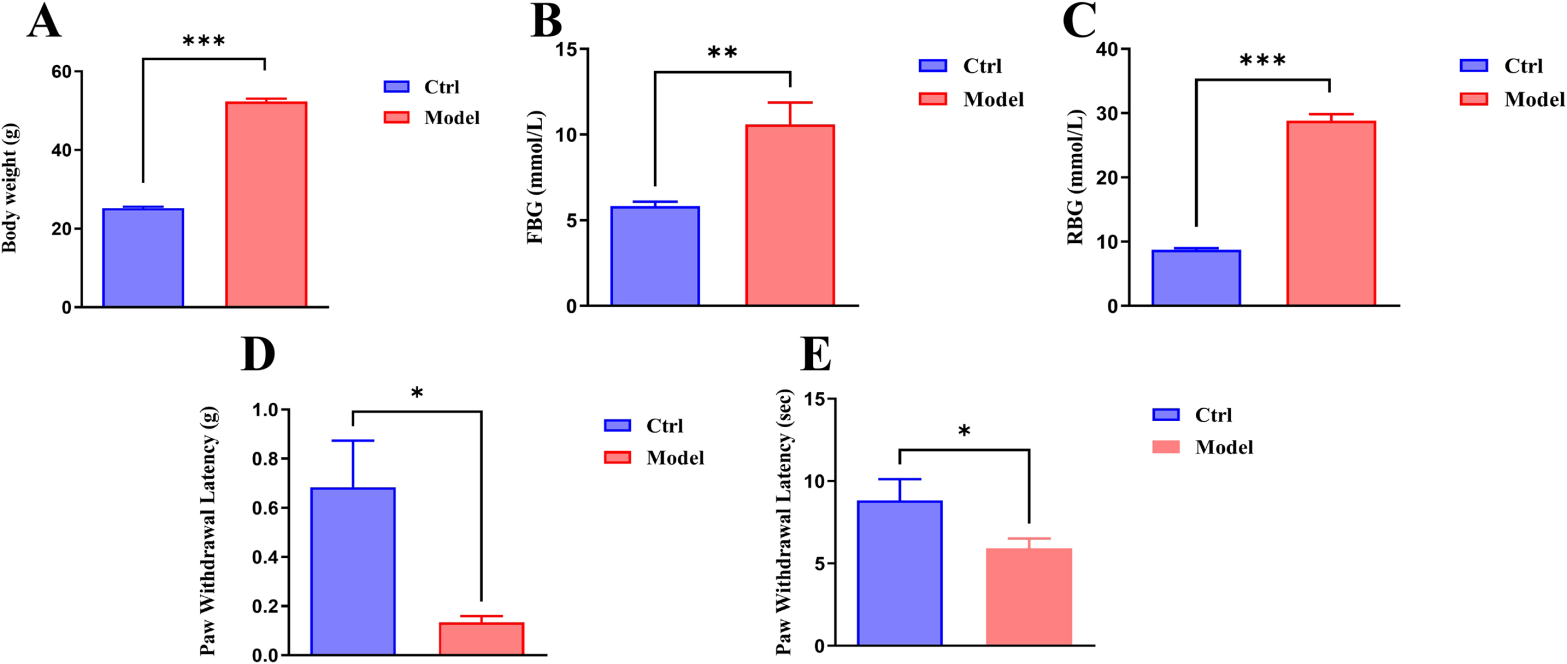
Spontaneous diabetic db/db mice exhibit increased body weight and blood glucose levels accompanied by reduced pain sensitivity. **(A)** Comparison of body weight **(g)** between Ctrl and Model mice; **(B)** Comparison of fFBG (mmol/L) levels between the two groups; **(C)** Comparison of RBG (mmol/L) levels between the two groups; **(D)** Behavioral test results of paw withdrawal latency (g) in response to mechanical stimuli in the two groups; **(E)** Behavioral test results of paw withdrawal latency **(sec)** in response to thermal stimuli in the two groups. *P<0.05, **P<0.01, ***P<0.001 indicating significant differences.

To further investigate the expression of the three key genes (HSPB1, KRT14, SFN) in DN, a spontaneous diabetic db mouse model was used as the DN model group (Model), with C57BL wild-type mice of the same sex, age, and genetic background serving as the control group (Ctrl). Then, the expression of HSPB1, KRT14, and SFN in the dorsal root ganglion tissue and spinal cord tissue was analyzed. The results showed that compared to the Ctrl group, the Model group exhibited significantly increased levels of Body weight (**Figure 7**), fasting blood glucose (FBG) (**Figure 7**), and random blood glucose (RBG) (**Figure 7**), while Paw withdrawal latency (g) (**Figure 7**) and Paw withdrawal latency (sec) (**Figure 7**) were significantly decreased. This indicates that our DN mouse model was successfully established.

**Figure 8:**
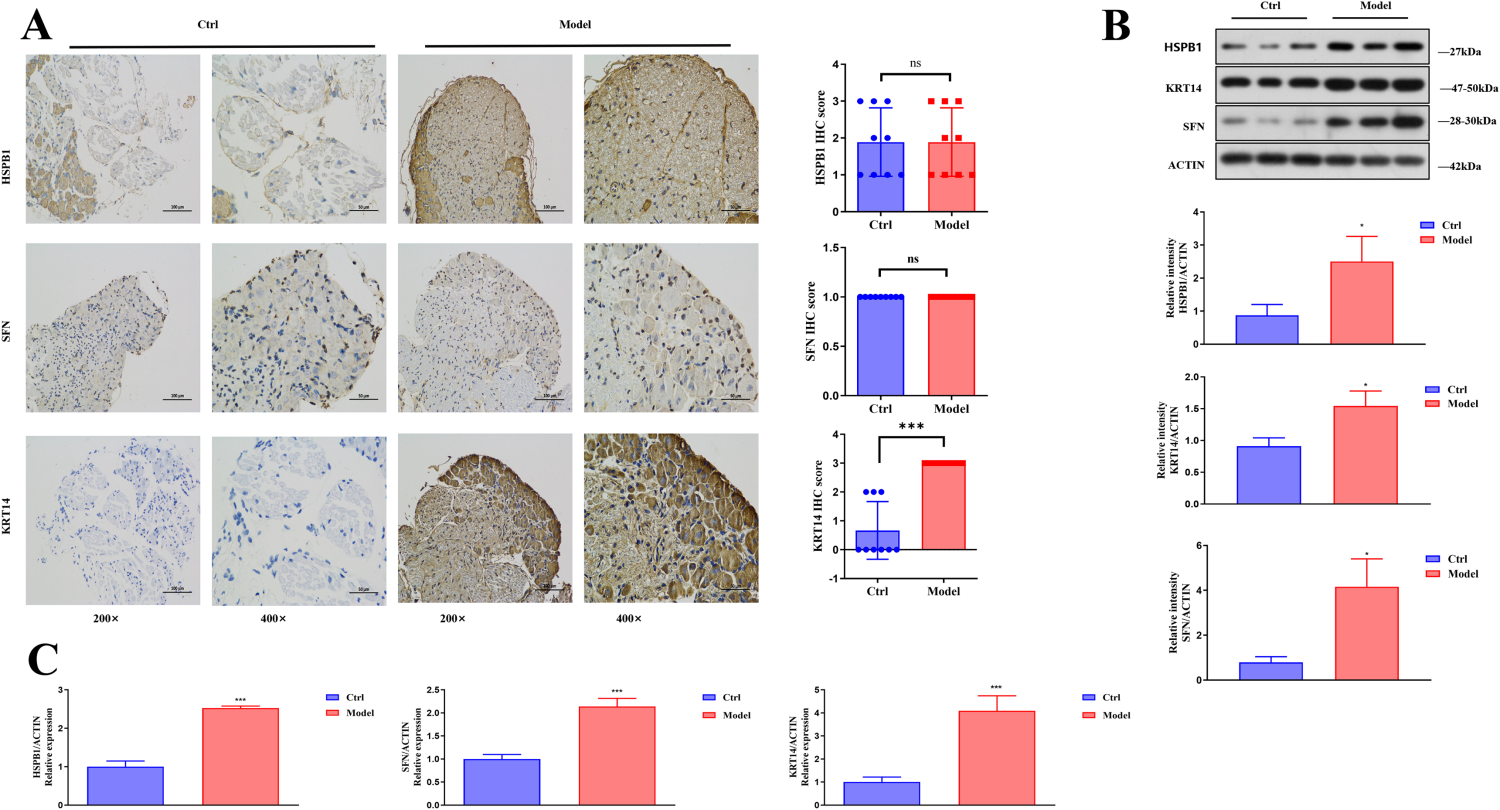
Upregulated expression of HSPB1, KRT14, and SFN in dorsal root ganglion and spinal cord tissues of diabetic db/db mice. **(A)** Immunohistochemical staining showing the protein expression levels of HSPB1, SFN, and KRT14 in dorsal root ganglion tissues from Ctrl and Model mice, with quantitative analysis of IHC scores presented in the bar graphs; **(B)** Western Blotting analysis of HSPB1, KRT14, and SFN protein levels in mouse spinal cord tissues, with relative protein expression quantified by normalizing to ACTIN; **(C)** qRT-PCR analysis of HSPB1, SFN, and KRT14 mRNA expression levels in mouse spinal cord tissues, with relative expression normalized to Actin. *P<0.05, ***P<0.001 indicating significant differences.

Subsequently, we examined the expression of HSPB1, KRT14, and SFN in the dorsal root ganglion tissue using immunohistochemistry. We found that compared to the Ctrl group, the levels of HSPB1 and SFN in the Model group showed no significant changes, while KRT14 was significantly elevated (**Figure 8**). Additionally, we detected the expression of HSPB1, KRT14, and SFN in spinal cord tissues using Western Blotting and qRT-PCR. The results indicated that compared to the Ctrl group, HSPB1, KRT14, and SFN were significantly increased in the Model group (**Figure 8B**). In conclusion, our *in vivo* animal experiment results showed that HSPB1, KRT14, and SFN were highly expressed in DN mice. These findings provided a new direction for the early diagnosis and treatment of DN.

## Discussion

DN is one of the most prevalent chronic complications associated with diabetes. While symptomatic treatments are available for DN, few therapeutic regimens effectively address the underlying causes. DN imposes substantial physical, psychological, and economic burdens, highlighting the urgent need for cost-effective and targeted therapies (26). In this context, we identified HSPB1, KRT14, and SFN as robust diagnostic biomarkers and key mediators of DN by integrating MR, Bayesian colocalization, RNA sequencing data, machine learning, bioinformatics analysis, and *in vivo* animal experiments, thereby providing critical insights for the treatment of DN and drug development.

Our study utilized the largest human plasma proteomics dataset to date, conducting MR analysis on plasma proteins to investigate their causal relationships with DN. We identified six proteins (HSPB1、SFN、KRT14、SPINT2、PPP1R14A、NOTUM) associated with a reduced risk of DN and one protein (DCXR) linked to an increased risk, with several involved in glucose metabolism (HSPB1, DCXR, SFN, NOTUM) (**Figure 2**). Among these candidates, HSPB1 have been previously documented to be associated with DN pathogenesis; clinical studies have confirmed elevated serum HSPB1 levels in DN patients, and its expression is positively correlated with the risk of distal symmetric polyneuropathy, the most common subtype of DN (27, 28). NOTUM showed negative associations with DN (29). To the best of our knowledge, no prior studies have reported the association of the remaining six genes (DCXR, SFN, KRT14, SPINT2, PPP1R14A) with diabetic neuropathy, and their potential regulatory roles in DN have not been elucidated. To identify potential therapeutic targets for DN, we performed integrative validation analyses. Since the identified proteins could only screen for 1–2 SNPs, heterogeneity and pleiotropy tests were not feasible, which complicated the application of sensitivity analysis for the robustness of the MR results. Consequently, we undertook the following study to verify the heterogeneity of the findings. Initially, we performed external validation of the MR data and identified that SPINT2, NOTUM, PPP1R14A, and DCXR have causal effects on DN. However, HSPB1, SFN, and KRT14 were not validated in future external datasets (**Supplementary Table 2**). A potential reason for this discrepancy is the variation in LD patterns across different populations or datasets, which may lead to biased effect estimates. The inability to conduct sensitivity analyses may have contributed to the failure of external validation. Bayesian colocalization evidence strongly supports the association between HSPB1, SFN, KRT14, SPINT2, DCXR, and DN (**Figure 2**). To validate the results identified through MR, we also utilized the GEO database. In the analysis of the GSE148061 dataset, significant differences in protein expression identified by MR were observed, with DCXR showing significantly higher expression in the DN group, while HSPB1, KRT14, and SFN exhibited significantly lower expression. Next, DN was divided into two subtypes, differential analysis of the MR-screened genes revealed that KRT14, NOTUM, SFN, and SPINT2 were significantly elevated in the cluster 2 group (**Figures 3**, **4**). We observed that MR identified proteins showed divergent expression between the GSE148061 dataset and cluster, likely due to sample heterogeneity (e.g., disease stage, age, and sex), which may have resulted in the misattribution of protein expression differences. To address this potential bias and avoid misattribution of protein expression differences, we integrated multiple complementary analytical strategies and *in vivo* validations to rigorously filter and verify the core candidate genes, ensuring the robustness of our final findings. This integrative approach identified genes with significant expression changes, which may play key roles in the pathogenesis of DN.

To elucidate the regulatory mechanisms of the identified genes in the human body with DN, this study conducted biological function and signaling pathway enrichment analyses on the DEGs from the GSE148061 dataset. GO analysis revealed significant enrichment in histone modification, methylation, epidermis development, and skin development (**Figure 3**). These findings underscore the importance of epigenetic modifications, consistent with previous research (30–32). Additionally, KEGG analysis indicated significant enrichment in the Wnt signaling pathway, Hippo signaling pathway, mTOR signaling pathway, and endocytosis (**Figure 3**). Prior studies have shown that the Wnt pathway contributes to neural injury repair (33). The Hippo signaling pathway, a critical regulator of cell growth, proliferation, and apoptosis, has been demonstrated to play a significant role in the regulation of diabetes and its complications (34). Furthermore, mTOR and its associated signaling pathways influence various metabolic parameters, including cellular metabolic homeostasis, insulin resistance, insulin secretion, stem cell proliferation and differentiation, pancreatic β-cell function, and programmed cell death, accompanied by apoptosis and autophagy (35). Research indicates that rapamycin, an mTOR inhibitor, when administered intrathecally, enhances the functional availability of dorsal root ganglia and alleviates STZ-induced hyperalgesia in rats (36). Collectively, these findings reveal that DEGs are implicated in epigenetic modifications, skin development, and complex regulatory networks.

To more accurately identify therapeutic targets for DN and their mechanisms of action, this study employed LASSO and RF for further analysis of the dataset. LASSO regression is typically used for variable filtering and mitigating the risk of overfitting. The binomial deviance method can be utilized to determine the optimal number of DEGs (37). RF excels in gene ranking and is highly suitable for managing high-dimensional data, constructing prognostic models, and assessing the importance of individual variables (38). In this study, the results obtained through the two machine learning methods identified HSPB1, KRT14, and SFN genes as potential therapeutic targets (**Figure 5**). This finding was subsequently validated in a DN mouse model through Western Blotting and qRT-PCR analysis of spinal cord tissues. The levels of HSPB1, KRT14, and SFN were significantly increased in DN mice. Immunohistochemical analysis indicated a significant increase in KRT14 levels in the DRG, while no notable changes were observed in HSPB1 and SFN (**Figure 8**). These discrepancies may stem from the distinct roles that these proteins play in various neural tissues or the spatiotemporal regulation of gene expression (transcription, translation, degradation). Notably, no published studies have investigated the expression of HSPB1 and SFN in the DRG of DN animal models; existing studies of HSPB1 in DN are restricted to clinical serum measurements (27, 28), with no evidence for its expression in specific neural tissues. These discrepancies may stem from the distinct roles that these proteins play in various neural tissues or the spatiotemporal regulation of gene expression (transcription, translation, degradation). The DRG and spinal cord exhibit divergent functional responses in diabetic neural injury. Our results of significantly upregulated HSPB1 and SFN in the spinal cord (**Figure 8B**) via Western Blotting and qRT-PCR have sufficiently confirmed their association with DN pathogenesis.

Among the three core genes screened in this study, HSPB1, also known as HSP27, is a molecular chaperone belonging to the small heat shock protein family. In patients with diabetic peripheral neuropathy, serum levels of HSPB1 are elevated (28). Distal symmetric polyneuropathy represents the most common form of DN. Logistic regression analysis indicates that serum HSPB1 levels are associated with a twofold increase in the odds ratio for distal symmetric polyneuropathy (27), corroborating our findings. KRT14, a member of the keratin family, is associated with the connective tissue of interfollicular epidermal cells in mammalian skin (39) and plays a role in the differentiation of keratinocytes (40). Studies have demonstrated that epidermal keratinocytes shift their function to act as sensors and transducers for both nociceptive and non-nociceptive sensations, closely interacting with intraepidermal nerve fibers within the neurocutaneous unit (41). KRT14 may influence neural effects by modulating keratinocyte differentiation. Additionally, KRT14 is overexpressed in the urine of bladder cancer patients and serves as a marker for bladder progenitor cells (42). *In vitro* experiments have confirmed that exosomes containing orbital mucosal periosteal Krt14+Ctsk+ cells significantly enhance the proliferation, migration, and angiogenic induction capabilities of human umbilical vein endothelial cells. Furthermore, these exosomes markedly increase the expression of osteogenic markers, indicating their potential to enhance osteogenic capabilities (43). SFN, also known as 14-3–3 sigma, functions as a cell cycle checkpoint protein. Aminopeptidase N (APN)/CD13 is identified as a potential fibroblast receptor for SFN. Research has shown that APN expression can be induced in dermal fibroblasts in the presence of keratinocytes or in response to keratinocyte-conditioned medium (44). Quantitative proteomic techniques have identified SFN as a potential biomarker for diagnosing perineural invasion in stage II colorectal cancer patients (45). In MCF-7 breast cancer cells, SFN mediates IGF-I-induced cell cycle progression (46). Notably, while HSPB1 has been well-documented to be associated with DN, the regulatory roles of KRT14 and SFN in DN pathogenesis have not been previously reported. Our study is the first to reveal the potential involvement of KRT14 and SFN in DN, and these findings expand the understanding of the molecular mechanisms underlying DN. Meanwhile, HSPB1, KRT14, and SFN represent a potentially promising therapeutic target for DN, and further research is warranted to decipher the mechanism through which they contribute to neurological injury.

Subsequently, we conducted GSVA and GSEA on HSPB1, KRT14, and SFN. HSPB1 is primarily enriched in the Wnt Signaling Pathway (**Figure 6**). Studies have demonstrated that modulation of the Wnt signaling pathway can ameliorate symptoms of DN, concurrently improving myelin sheath lesions, promoting nerve regeneration, and activating Schwann cells (33). Intrathecal (10 and 30 μM) and intraperitoneal (10 mg/kg) injections of isoquercitrin (ISQ) significantly downregulated the expression of Wnt/β-catenin pathway proteins in diabetic rats, resulting in an improved behavioral pain threshold. This indicates that inhibition of the Wnt/β-catenin signaling pathway possesses neuroprotective potential in DN (47). KRT14 may exert its effects through pathways such as ERK1 and MAPK cascades. Gabapentin and duloxetine have been shown to inhibit ERK1/2 phosphorylation in the extracellular signaling of mouse spinal cord. Furthermore, they prevent the development of neuropathic-like pain behaviors induced by oxaliplatin and paclitaxel by inhibiting ERK1/2 activation in the mouse spinal cord (48). RNA sequencing combined with quantitative PCR and Western Blotting analyses elucidated that high glucose activates the MAPK/ERK signaling pathway in human umbilical vein endothelial cells. In STZ-induced rats, DN is associated with increased p38-MAPK expression in peripheral nerves (49). Additionally, activated MAPK signaling can promote oxidative stress and neuronal degeneration (50). SFN may influence the Insulin Signaling Pathway and B Cell Receptor Signaling Pathway. Insulin provides critical support to neurons and axons, and abnormalities in insulin signal transduction in peripheral neurons are linked to the development of DN. Impaired insulin receptor signaling in peripheral nerves has been observed during the early stages of altered nociception in STZ-induced diabetic rats (51). The intrathecal delivery of low-dose insulin (0.1-0.2 IU daily) to neurons improved and reversed the slowing of motor and sensory conduction velocities in rats with STZ-induced diabetes (52). In previous bioinformatics analysis studies, compared with healthy controls, DN was mainly enriched in Apoptosis and the B Cell Receptor Signaling Pathway (53). Based on previous research findings and our discoveries, this study reveals the potential mechanisms of action of HSPB1, KRT14, and SFN, providing an important foundation for future research on DN.

This study integrates MR with bioinformatics data analysis to explore the critical roles of HSPB1, KRT14, and SFN in DN, validating these findings through *in vivo* animal models. Although MR analysis identified HSPB1, KRT14, and SFN as associated with reduced DN risk, our transcriptomic and *in vivo* data demonstrate their upregulation in DN samples and animal models. This apparent contradiction is biologically plausible, as these upregulations likely reflect compensatory or protective responses to diabetic neural injury. Furthermore, their altered expression in disease states suggests active participation in key signaling pathways underlying DN pathophysiology. Therefore, despite their association with reduced disease risk, these genes represent valid candidates for further investigation as potential regulatory or restorative therapeutic targets. However, several limitations must be acknowledged. Firstly, the GWAS data were from European ancestry individuals, which may limit the generalizability of our findings to other ethnic groups. Further investigations are warranted to validate our findings across different ethnic and racial populations. Moreover, tissue-specific protein data are scarce, so this study focused mainly on plasma proteins. Although plasma biomarkers are often advantageous for disease screening and diagnosis due to their low invasiveness and ease of accessibility, assessing protein roles in other tissues, particularly neural tissues, could yield additional insights into the pathogenesis of DN. Secondly, despite employing the largest available GWAS dataset for DN, the sample size may increase bias and limit statistical power. Therefore, further research is essential to enhance and broaden the sample size for replicating these findings. Although MR reduces confounding, complete elimination of residual confounding is not guaranteed. Additionally, the shortcomings of sensitivity analysis raise concerns regarding the stability of the results. Future large-scale epidemiological studies are needed to validate plasma protein levels as DN biomarkers. While this study provides a preliminary framework for understanding the action patterns of risk-reducing genes and their upregulation in DN, their specific mechanisms of action remain to be elucidated. Future functional experiments, such as gene knockout or overexpression, are warranted to clarify their precise roles in DN pathophysiology, providing further support for their potential as therapeutic targets.

In this research, MR, Bayesian colocalization, bioinformatics, various machine learning algorithms, and *in vivo* animal models were comprehensively employed to identify HSPB1, KRT14, and SFN as potential biomarkers for DN. The identification of these proteins contributes to advancing research into the etiology of DN, clinical diagnosis, and the exploration of potential therapeutic agents. However, additional experimental and clinical studies are required to fully elucidate the role of these proteins in DN development and to evaluate the utility and efficacy of these candidate drugs, thereby confirming the present results.

## Data Availability

The original contributions presented in the study are included in the article/**Supplementary Material**. Further inquiries can be directed to the corresponding author/s.
